# Acid selective pro-dye for cellular compartments

**DOI:** 10.1038/s41598-019-50734-8

**Published:** 2019-10-25

**Authors:** Barbara Czaplińska, Katarzyna Malarz, Anna Mrozek-Wilczkiewicz, Robert Musiol

**Affiliations:** 1 Institute of Chemistry, University of Silesia in Katowice, 75 Pułku Piechoty 1, 41-500 Chorzów, Poland; 2 A. Chełkowski Institute of Physics and Silesian Center for Education and Interdisciplinary Research, University of Silesia in Katowice, 75 Pułku Piechoty 1, 41-500 Chorzów, Poland

**Keywords:** Cellular imaging, Organic chemistry

## Abstract

A novel pro-dye approach for the acid-selective staining of the subcellular compartments for better permeability and selectivity was applied. The designed sensor has suitable physicochemical properties such as a large Stokes shift and a long-lived intracellular fluorescence. The Schiff base fragment was used for the acid-sensitive release of a fluorophore without affecting the overall stability of the biological systems. Due to the presence of an imine bond in its structure and its unique fluorescent properties, it can be presented as a “pro-dye” for acidic structures such as lysosomes. As a result of an imine bond cleavage, a new fluorescent compound is released, whose substantially shifted excitation and emission wavelengths enable a more selective and effective imaging of lysosomes and endosomes. The presented report provides the chemical, physicochemical and optical profiles as well as biological assays and theoretical calculations.

## Introduction

A single cell reflects the astonishingly complex landscape of the acidic and basic compartments. From the slightly basic mitochondria, pH scales up to a thousand times higher concentration of hydronium ions in the endosomes and lysosomes. The latter are small membrane-enclosed organelles, which are considered to be the digestive system of a cell. Their typical functions are connected with the sequestration and degradation of all types of macromolecules^1^. In response to various stimuli, they become a source of the apoptotic factors^2^. Low lysosomal pH (4–5) is necessary for maintaining the activity of the proteolytic enzymes such as hydrolases. Importantly, this is a rather fluent equilibrium, which causes the cellular compartments to be more or less acidic and changes the situation during the lifetime of a cell according to metabolic processes. In fact, changes in pH are linked with endo- or exocytosis – the life cycle, particularly oxphos and glycolysis, as well as with apoptosis or muscle contractions, thus suggesting the possibility of its exploitation as a diagnostic tool in medicine, e.g. to distinguish between normal and cancer cells^3^. Unfortunately, measuring pH remains one of the more demanding tasks in a living biological environment, despite its simplicity in more typical inorganic analytical systems. When considering a single living cell, size and fragility become the main problems. Fluorescent microscopy and related techniques remain the best choices for living cells. The availability of fluorescent probes for the different subcellular compartments and applications is growing, although there are still some confusing gaps in this field^4^. Besides their fluorescent properties, the main problems that have to be addressed in designing fluorescent probes are their selectivity, signal quality and neutrality to the environment that was being measured. In this regard, the commercially available probes such as acridine orange or LysoTracker are not free from drawbacks such as a lack of specificity or photobleaching. Rhodamine, which is connected with the N-linked glycans, was recently proposed by Yapici *et al*. for selectively targeting lysosomes^5^. Although such an approach results in excellent usable probes, it also has a risk of overfitting. From a diagnostic point of view, it is even more important to combine selectivity with the ability to detect changes beyond these organelles. For example, leakages of the lysosomal content or endocytosis are other essential aspects that require less selectivity.

Styrylquinolines are an appealing scaffold for designing cellular probes and dyes as well as providing good synthetic availability and a facility for desirable fluorescent properties. Our research group is strongly interested in synthesizing and investigating quinoline derivatives^6–8^. Simple styrylquinolines have physicochemical properties that are promising for biological systems in terms of their fluorescent sensors^9^. For instance, Staderini *et al*. presented a 4-aminostyryl derivative of 6-methylquinoline with potential therapeutic and diagnostic properties against Alzheimer’s disease due to its ability to stain Aβ amyloid plaques^10^. Another good example is a styrylquinoline dye with the dipicolyamine moiety, which demonstrates a multicolor fluorescence depending on the addition of different metal ions^11^.

The low pH in lysosomes is a distinguishing feature that can be exploited in designing selective fluorescent probes. Nevertheless there are only few attempts to exploit this feature^12–14^. A quinoline conjugated with benzothiazole moiety (BTVQ) has been used as pH specific dye for lysosomes^15^. This probe has large Stokes shift and pKa of 3.5 but low wavelength of absorption maxima and reduction of fluorescence in low pH are drawbacks. Taking this into account, we designed a new compound based on a styrylquinoline scaffold with an imine bond in its structure. Due to its character, the C = N bond seems to be particularly useful in this regard. It has a relatively good metabolic stability, which permits hours of incubation. On the other hand, it can easily be broken in acidic conditions^16^. With this in mind, we designed a Schiff base that can serve as a “pro-dye”, a carrier that releases the proper fluorescent dye in the acidic environment of lysosomes. Further, we present a comprehensive exploration of the newly synthesized compound **BC15**, including the chemical, physicochemical and optical profiles as well as biological assays and theoretical calculations.

## Results

### Synthesis and characterization

**Figure 1:**
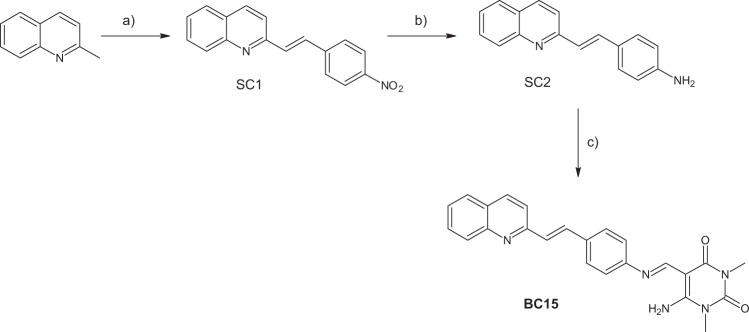
The synthetic pathway of compound **BC15**. Reagents and conditions: (**a**) 4-nitrobenzaldehyde/Ac_2_O, (**b**) SnCl_2_/EtOH, (**c**) 6-amino-1,3-dimethyl-2,4-dioxo-1,2-3-4-tetrahydropyrimidine-5-carbaldehyde /EtOH, microwave conditions 50 W, 80 °C, 20 min.

Our synthetic approach is depicted in Fig. 1. The investigated compound was prepared by a three-step reaction: (1) the synthesis of a nitrostyrylquinoline derivative in a simple, efficient reaction between quinaldine and p-nitrobenzaldehyde in acetic anhydride conditions. This reaction has previously been reported by our research team as an easy and convenient way to obtain the styryl derivatives that are based on quinoline scaffold^8,17^; (2) a reduction of a/the nitro group to a/the amino group using anhydrous tin (II) chloride SnCl_2_ as the reducing agent in mild conditions of dry ethanol and (3) a quick and efficient reaction between an amino derivative and 6-amino-1,3-dimethyl-2,4-dioxo-1,2-3-4-tetrahydropyrimidine-5-carbaldehyde under microwave conditions.

### Spectroscopic properties

**Figure 2:**
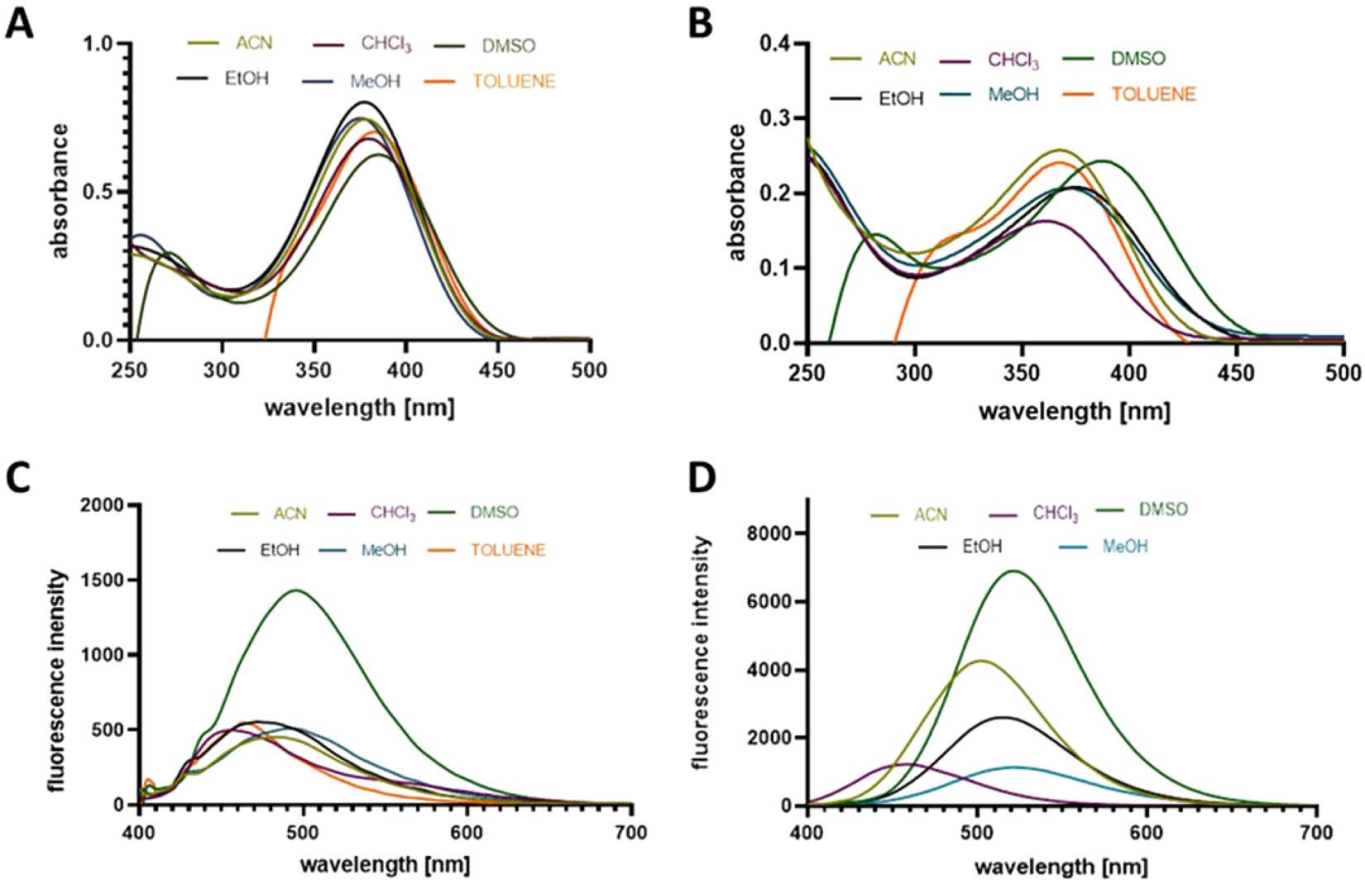
Absorption spectra of **BC15** (**A**) and **SC2** (**B**) and the emission spectra of **BC15** (**C**) and **SC2** (**D**) in solvents with different polarities. The absorbance in toluene is suppressed du to intermolecular interactions (π stacking).

To obtain the full spectroscopic characteristics, the measurements were performed in various solvents with different polarities and the ability to create hydrogen bonds. The performed studies gave satisfactory results, which are presented in Table 1 and Fig. 2. Compound **SC2** exhibits similar absorption and emission properties to compound **BC15**. All absorption spectra have their maxima over 360 nm, which is beneficial in cell imaging, because excitation with shorter wavelengths may cause autofluorescence from aromatic aminoacids (e.g. tryptophan), flavins or NADH that exhibit emission in blue region^18^. Moreover,the shorter wavelengths have enough energy to cause some mutagenic effects.

**Table 1:** Spectroscopic properties of compounds **BC15** and **SC2**.

Solvent (Snyder polarity index^a^)	BC15			SC2		
	absorption [nm]	emission [nm]	Stokes shift [nm/cm^−1^]	absorption [nm]	emission [nm]	Stokes shift [nm/cm^−1^]
toluene (2.3)	385	460	75/4235	368	—	74/4549
chloroform (4.4)	380	465	80/4576	362	457	95/5742
acetonitrile (6.2)	378	470	92/5178	368	502	134/7253
ethanol (5.2)	380	478	98/5395	375	514	139/7211
methanol (6.6)	378	492	114/5395	372	522	150/7211
DMSO (6.5)	386	495	109/5705	388	522	134/6616
EtOH/water + HCl	degradation of the compound			470	606	136/4775

^a^according to ref.^49^.

Moreover, it was easily noticeable that the absorption spectra of **SC2** are more sensitive to changes in solvent’s polarity than the spectra of **BC15**. One can observe a shift of the maxima from 368 nm in toluene to 388 nm in DMSO. For DMSO and toluene significant drop op absorbance can be seen in the range below 250 and 350 nm respectively. This is an effect of non-covalent interactions as e.g. in aromatic solvent (toluene) which can form specific intermolecular interactions with the dye molecules (as π stacking) which can result of observable drop of absorbance. Similar effects were reported also by Zheng *et al*.^19^ As a consequence both SC2 and BC15 have no maxima below 280 nm in those solvents. The emission spectra, however, are much more dependent on the polarity because the values of the fluorescence maxima vary more significantly for both compounds. The shortest wavelength was observed for **SC2** in chloroform and amounted to 457 nm (blue fluorescence). Then, it redshifted gradually along with the polarity of a solvent and had the largest batochromic shift in DMSO (522 nm, green fluorescence). Conversely, the values of the emission maxima for the **BC15** ranged from 460 nm to 495 nm and emitted blue and blue-green fluorescence. These characteristic changes have been described as positive solvatochromism^20^. Changes in the electronic transitions that are caused by the interactions of solvents may be also the reason for such large Stokes shifts, especially in polar, protic solvents. The highest values were 150 nm (7,724 cm^−1^) for **SC2** and 114 nm (6,130 cm^−1^) for **BC15** in methanol. These considerable solvent-dependent changes in the emissions can be explained on the basis of the electron density distribution in the ground and excited states. The visualization of the frontier molecular orbitals (Supplementary Fig. S1) that are involved in the main electronic transitions enabled any changes in the electron distribution that occurred in a molecule after photoexcitation to be analyzed. In both cases (compound **SC2** and **BC15** as well) one can observe the charge transfer character of the transitions. HOMO is mainly localized on the phenyl moiety (SC2) due to the presence of the strong electron-donating group -NH_2_. On the other hand, the electron density of LUMO is shifted toward the quinoline ring, which is known to be an acceptor unit. The similar effect can be observed with BC15 compound.

When it comes to emission efficiency, heterocyclic compounds containing a nitrogen atom, low-lying n→π* transition causes the relatively low fluorescence intensity in non-polar solvents. In protic solvents such as alcohols, the lowest-lying state n→π* can be inversed with the π→π* state due to the formation of hydrogen bonds (HB) between the nitrogen atom and solvent molecules. In this case however, we observed opposite effects, which suggest that different interactions (presumably connected with the 6-amino-1,3-dimethyluracil unit) are involved in the stabilization/destabilization of the excited state. In DMSO which is aprotic but polar solvent the fluorescence is considerably higher for both compounds that were tested. To describe the most probable reason for this effect, besides the commonly known Snyder’s solvent polarity index, we additionally took under consideration Kamlet – Taft theory^21^, which involves hydrogen bonding as important factors influencing fluorescent properties. The theory introduces two main parameters describing the hydrogen bond accepting (β) and donating (α) properties of solvent (data summarized in Table S1 Supporting Information). In this regard DMSO as an pure acceptor of HB (β = 0.76, α = 0) interacts with the amino group (which acts as hydrogen bonds donor). Such bonding facilitates intramolecular charge transfer and favoring ICT emission. On the other hand, alcoholic solvents that can act as HB acceptors and donors. In this case, forming of HB between nitrogen atom of amino group and hydrogen of protic solvent results in decreasing of accessibility of nitrogen lone pair for ICT process. Thus, the possibility of nonradiative process is smaller in DMSO, since the ICT state is better stabilized in solvents with hydrogen bond accepting ability. What is more, polar solvents and formation of the HB by amino group suppress its tendency to rotation which could provide a nonradiative pathway of S_1_ state deactivation. Thus specific interactions like HB and their energies in the excited state also play a vital role in solvatochromic properties and deactivation of ICT excited state^22,23^. Similar effects were reported for other aza-heterocycles^24–26^.

### Water and pH dependence of the spectroscopic properties

**Figure 3:**
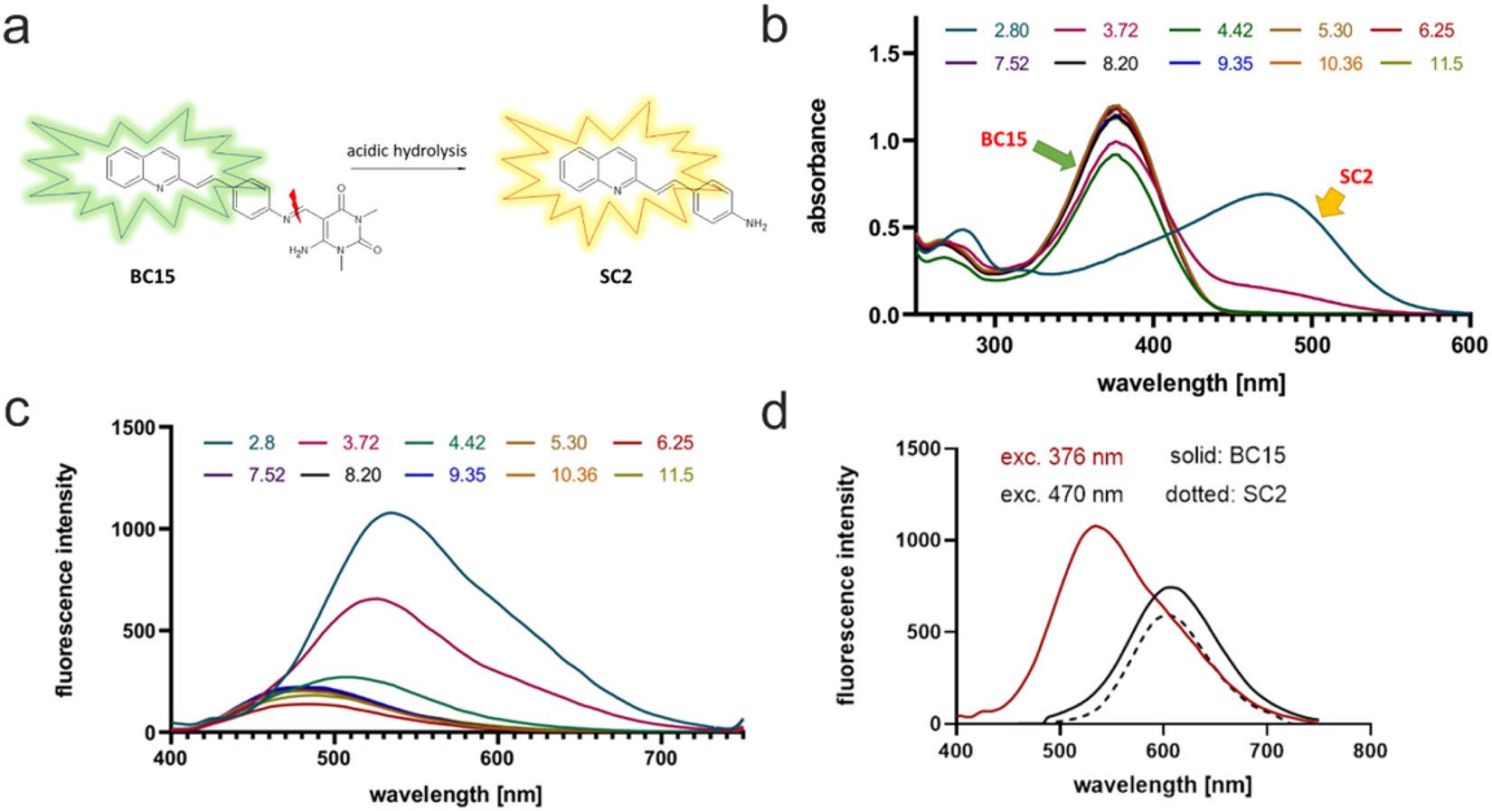
Pro-dye strategy for detecting low pH in lysosomes and endosomes. (**a**) Acid hydrolysis of the dye. (**b**–**d**), spectroscopic characteristics of the dyes. (**b**) Absorbance of the pro-dye **BC15** and fluorophore **SC2**. Below pH = 4, decomposition and the formation **SC2** begins, which later gets protonated in pH below 3, thereby resulting in a more red-shifted absorbance. This second maxima of absorbance can be used for the excitation of the released dye solely as is presented in (**d**). (**c**) pH-dependent changes in fluorescence for the dye. During acidification, no changes were observed until pH 5.3 when the maxima shifted to the higher wavelengths. (**d**) Comparison of the **BC15** and **SC2** fluorescence. Excitation with 376 nm only emitted a green fluorescence while excitation with 470 resulted in an orange fluorescence only from the acidic organelles.

Taking into consideration the potential applications of the newly synthesized compound (**BC15**) as an acid-selective “pro-dye”, we decided to investigate its spectroscopic properties in a water environment. The main goal of this research was to obtain a compound that has an imine bond cleavage in acidic conditions, but that is stable in a cytosol or other organelles. Therefore, our attention was focused on the possibility of inducing a hydrolysis process in aqueous conditions^27^. For that purpose, we prepared a set of samples that contained 25% water and 75% of ethanol which were measured using the HPLC method after 5, 15, 30, and 60 minutes to determine the progress of decomposition at given time. The results of this study are presented in Supplementary Fig. S2. The pH-dependence of fluorescence is a crucial issue for such a dye. To determine this, we performed a number of spectra measurements in water/EtOH solutions of various pH values. The obtained results are presented in Table 2 and Fig. 3.

**Table 2:** Spectroscopic properties of compound BC15 depending on the pH of the solution.

pH	Absorption [nm]	Emission [nm]
2.80	470	536
3.72	376/470	527
4.42	376	512
5.3	376	488
6.25	376	488
7.52	376	488
8.2	376	488
9.35	376	488
10.36	376	488
11.50	376	488

After a comprehensive analysis of the obtained results, it was determined that the compound exhibited a relatively stable fluorescence in a pH range between 12 and 5. All of the spectra in this range had a similar intensity of emission and a constant wavelength of the maxima of the bands. Below pH = 5, the maxima of the bands showed a progressive redshift (to a wavelength of 536 nm) and the fluorescence intensity increased about five-fold. In the case of the absorption spectra, the situation was similar – in the range of pH 12 and 5, there were no relevant changes. In more acidic conditions, however, a new significantly redshifted band (470 nm) appeared as a signal from the protonated form of decomposition product (**SC2**) as is presented in Fig. 3b,c. These two phenomena are particularly appealing for the proposed imaging application because they increase the selectivity and the signal to noise ratio. It is worth mentioning that all of the emission spectra that were measured in this experiment were excited at the same wavelength −376 nm. In view of the changes that occurred at low values of pH, we measured the additional spectra when the probe was excited with a signal at 470 nm (Fig. 3d), thereby obtaining emission spectra with a maximum at 606 nm, which corresponds to orange light.

### Biological activity and cellular imaging

**Figure 4:**
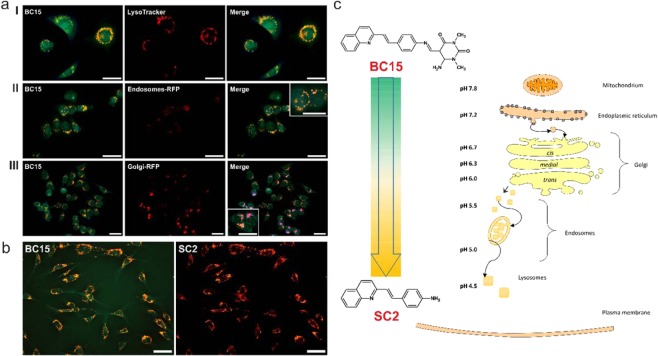
Fluorescence images of the HCT 116 cells that had been incubated with **BC15** (25 µM) for 2 h at 37 °C. (**a**) **BC15** was excited by a DAPI (UV-2A filter; excitation wavelength 330–380 nm; barrier filter BA420). The images show a co-localization with specific organelle trackers: lysosomes (I), endosomes (II) and the Golgi apparatus (III). First and second panel – fluorescence of the **BC15** or specific dye alone, last panel – merged (red-orange indicates colocalization of **BC15** with specific-organelle tracker, magenta indicates area where no colocalization was observed). (**b**) Fluorescence images of the NHDF cells that had been incubated with **BC15** (25 µM for 2 h at 37 °C) excited by a DAPI (UV-2A filter; excitation wavelength 330–380 nm; barrier filter BA420) (**BC15**) and (B-2A filter; excitation wavelength 450–490 nm; barrier filter BA520) (**SC2**). Scale bars indicate 50 µm. (**c**) Representation of the **BC15** decomposition in the acidic organelles.

In the proposed applications, the inactivity toward the biological systems is of special importance. In order to evaluate this, we performed antiproliferative tests on a human colon cancer cell line (HCT 116) and normal human fibroblasts (NHDF) (Supplementary Table S2). In general, within the concentrations that are useful for staining, the tested compounds demonstrated no cytotoxicity against the cells that were investigated. The absorption profile of **BC15** enabled excitation with DAPI wavelength (UV-2A filter –excitation 330–380 nm) (Fig. 2a) or with a B-2A filter (excitation wavelength 450–490 nm) as a lower-energy source of excitation. After excitation at a shorter wavelength, dual-color fluorescence was observed. That feature was subsequently examined in the HCT 116 cells using distinct specific-organelle markers for lysosomes (LysoTracker® Red DND-99), endosomes (CellLight® Early Endosomes-RFP, BacMam 2.0), the Golgi apparatus (CellLight® Golgi-RFP, BacMam 2.0), the endoplasmic reticulum (ER-Tracker™ Red BODIPY® TR Glibenclamide) and mitochondria (MitoTracker Orange) (Fig. 4 and Supplementary Fig. S3). As a result of those experiments, it was concluded that compound **BC15** tended to accumulate in the membranous structures and their associated organelles as mitochondria and the endoplasmic reticulum, thus emitting a green fluorescence (Fig. S3 Supplementary Information). The orange fluorescence was attributed to the protonated product of **BC15** decomposition (**SC2**), which occurred mainly in the acidic organelles along the endocytic pathway where the pH values decreased from ~5.5 in the early endosomes to ~5.0 in the late endosomes and then decreased to ~4.5 in lysosomes (Fig. 4a)^4,28^.

Dual fluorescence make it difficult to analyze micrographs from co-localization experiments. Typical lysosome selective dyes interfere with green or orange fluorescence of **BC15**/**SC2**. To overcome this obstacle and confirm selectivity of our dye we have performed a quantitative assessment of protonated **SC2** co-localization with organelle-specific trackers using the Pearson and Manders methods. The correlation coefficient of each merged image was calculated with ImageJ software^29^ and the data are summarized in Table S3 (Supplementary Information). Based on the calculated data, lysosomal co-localization was characterized by high Pearson’s and Manders’ correlation coefficients, more than 0.6 (Table S3). On the other hand, we also observed a slight signal from **SC2** in the Golgi apparatus (Fig. 4a). The Pearson’s and Manders’ co-localization coefficients were 0.371 and 0.352, respectively. This observation may be because the biogenesis of the elements of the endocytic pathway occurs in the Golgi network^30^.

As was mentioned above, after excitation by UV-2A filter – wavelength 330–380 nm, we observed a dual fluorescence that originated from the presence of two different species in the HCT 116 cells. Compound **BC15** penetrated into almost all of the membranous organelles, while the protonated product of **BC15** decomposition (**SC2**) was only observed in the acidic compartments. In order to selectively obtain an image of **SC2**, a lower-energy source of excitation (450–490 nm) can be used. In this case, only the protonated form of **SC2** could be excited and emitted an orange fluorescent signal in the cells (Fig. 4b).

## Discussion

As was mentioned above, simple styrylquinolines with an amine group may be desirable as dyes for specific organelles. Their structure enables them to be derivatized, which then permits the straightforward control of their parameters, e.g. acidity. Their relatively lipophilic structure is also an advantage for permeability and accumulation. Unfortunately, despite its good spectroscopic parameters, 4-aminostyrylquinoline **SC2** was not able to fully penetrate the membranes, which resulted in a poor resolution and dim micrographs. Apparently it was not able to enter the lysosomes by itself as is presented in Supplementary Fig. S4, in which the HCT 116 cells that had been incubated with **SC2** showed a light blue-green fluorescence without a distinguishable signal. To overcome this problem, we designed several derivatives (data not shown) by converting the amino group into an imine group (Schiff base). This strategy offered several advantages, among which, the increase in lipophilicity and a simple adjustment of the selectivity are the most desirable. Schiff bases are known for their poor stability in aqueous solutions. They dissociate in acidic and basic conditions, which makes them relatively more stable in neutral pH^31^. In our experiments, the derivative of 2,4-dioxopyrimidine was the most effective stability enhancer. The resonance effects in this structure were strong enough to ensure a good stability in the neutral pH along with a ready dissociation in more acidic media. The spectroscopic properties of **BC15** appeared to be promising for such applications, in particular, its large Stokes shift and absorbance maxima, which permit excitation with DAPI filter. Excitation wavelengths that are shorter than 350 nm carry enough energy to destroy the complex protein structure, and therefore, dyes that require a high-energy excitation source are unsuitable for use in long-term experiments^32^. Additionally, excitation wavelengths above 360 nm enable the autofluorescence from naturally occurring molecules, whose excitation profiles embrace higher-energy wavelengths such as 270 nm (tyrosine)^33^, 280 nm (tryptophan)^34^ or 340 nm (NAD(P)H)^35^, to be avoided. Styrylquinolines can be toxic for eukaryotic cells^36,37^. However, in the case of **BC15** and **SC2**, the lack of substituents ensures low cytotoxicity in the usable concentrations^8,38^. Incubation with **BC15** or **SC2** for 72 h did not affect the viability of either the normal or cancerous cells. On the other hand, the lipophilic nature of the dye enabled fast penetration and good accumulation in membranous organelles which was observed after 1 h of incubation. The combination of large Stokes shifts, absorption over 370 nm and the good fluorescence profile in aqueous media indicates that the investigated compound (**BC15**) can be used as a molecular probe for biological staining. Additionally, the presented relationships between its fluorescent properties and pH indicate the possibility of also using the compound as a lysosomal pro-dye. A comprehensive investigation of the newly synthesized compound revealed several features that indicate that it can also be used as a biological probe for imaging acidic organelles (lysosomes) or for controlling intracellular pH alterations. **BC15** exhibited a very good excitation/emission profile, which enabled its safe injection into cells. Moreover, due to dual-color fluorescence and the possibility of excitation at two different wavelengths, we were able to adjust the conditions of an experiment to our preferences. The fluorescent signal was stable without any significant photobleaching, which enabled the cells to be imaged over extended periods of time. To confirm this statement we performed experiment in which cells treated **BC15** were exposed for the microscope irradiation (DAPI filter − 365 nm) during 1 hour. After that time we observed decrease in the fluorescence signal at the level of 25% (Supplementary Fig. S5). In addition, the compound exhibited a large Stokes shift, a lack of biological activity and stability in an aqueous environment. Experiments with the HCT 116 and NHDF cell lines indicated that **BC15** can be used in both healthy cells and cancer cells. Therefore, the newly synthesized compound constitutes a potential tool that can be used to investigate changes in the lysosomal environment inside a cell.

## Experimental

### Materials and basic measurements

All of the reagents were obtained from Sigma-Aldrich (St. Louis, MO, USA). The TLC tests were done on aluminum-backed silica gel 40 F254 silica gel plates (Merck, Darmstadt, Germany) under illumination with UV light (254 nm and 365 nm). The melting points are uncorrected and measured on an Optimelt MPA-100 apparatus (SRS, Stanford, CA, USA). The structures of the newly obtained compounds were confirmed using NMR spectroscopy. All of the NMR spectra were recorded on a Bruker AM-series with deuterated DMSO-d6 as the solvent (Bruker BioSpin Corp., Rheinstetten, Germany). The working frequency is given for each compound. Chemical shifts were measured against the internal standard and are given in ppm (δ), Si(CH_3_)_4_. Mass spectra were performed using a WATERS LCT Premier XE system (high resolution mass spectrometer with a TOF analyzer). The HPLC experiments were performed using an Agilent 1260 infinity II with FLD and DAD detectors.

### Synthesis and characterization

#### Synthesis of the starting material

*Step 1: 2-[(E)-2-(4-nitrophenyl)ethenyl]quinoline (SC1):* The synthesis was performed according to the procedure described in^8^. The quinaldine (10 mmol) and 4-nitrobenzaldedhyde (10 mmol) were mixed in acetic anhydride and heated for 20 h at 130 °C. The excess solvent was evaporated *in vacuo* and the obtained solid was purified using column chromatography with dichloromethane as the eluent. The product that was obtained was a bright yellow solid with a yield of 73% and a melting point of 167 °C. ^1^H NMR (400 MHz, DMSO) δ 8.41 (d, *J* = 8.6 Hz, 1H), 8.27 (d, *J* = 8.6 Hz, 2H), 8.02 (dd, *J* = 8.2, 4.2 Hz, 3H), 8.00–7.90 (m, 3H), 7.78 (t, *J* = 7.8 Hz, 1H), 7.73 (d, *J* = 16.4 Hz, 1H), 7.60 (t, *J* = 7.4 Hz, 1H). ^13^C NMR (126 MHz, DMSO) δ 155.27, 148.11, 147.36, 143.49, 137.24, 133.63, 132.22, 130.52, 129.30, 128.67, 128.35, 127.77, 127.17, 124.53, 120.82.

*Step 2: 4-[(E)-2-(quinolin-2-yl)ethenyl]aniline (SC2):* In a round-bottomed flask, compound **SC1** was placed in ethanol together with anhydrous SnCl_2_ at a molar ratio of 1:5. The mixture was heated for 2 h at a temperature of 70 °C under nitrogen conditions. Then, the mixture was cooled, placed in a flask with ice and neutralized with a 5% solution of sodium bicarbonate NaHCO_3_. Next, extraction with ethyl acetate was performed and the organic layer was washed with brine and dried over anhydrous sodium sulfate Na_2_SO_4_. The product that was obtained was a red solid with a yield of 61% and a melting point of 174 °C. ^1^H NMR (400 MHz, DMSO) δ 8.26 (d, *J* = 8.6 Hz, 1H), 7.91 (dd, *J* = 14.2, 8.3 Hz, 2H), 7.77 (d, *J* = 8.6 Hz, 1H), 7.69 (dd, *J* = 17.8, 11.8 Hz, 2H), 7.50 (t, *J* = 7.4 Hz, 1H), 7.42 (d, *J* = 8.3 Hz, 2H), 7.13 (d, *J* = 16.2 Hz, 1H), 6.61 (d, *J* = 8.3 Hz, 2H), 5.54 (s, 2H). ^13^C NMR (126 MHz, DMSO) δ 157.03, 150.39, 148.22, 136.54, 135.54, 130.07, 129.21, 128.81, 128.18, 127.10, 125.95, 124.22, 123.28, 120.06, 114.33. ^13^C NMR (126 MHz, DMSO) δ 157.03, 150.39, 148.22, 136.54, 135.54, 130.07, 129.21, 128.81, 128.18, 127.10, 125.95, 124.22, 123.28, 120.06, 114.33.

*Step 3: 6-amino-1*,*3-dimethyl-5-[(E)-N-{4-[(E)-2-(quiinolin-2-yl)ethynyl]phenyl} carboxy - imidoilo]-1*,*2*,*3*,*4-tetrahydropirymidyno-2*,*4-dion* Compound SC**2** and 6-amino-1,3-dimethyl-2,4-dioxo-1,2-3-4-tetrahydropyrimidine-5-carbaldehyde were placed in a 15 mL tube at a ratio of 1:1. Then, 5 mL of ethanol and three drops of acetic acid were added. The mixture was exposed to microwave irradiation for 20 minutes at 50 W and 83 °C. The obtained compound was filtered, washed with ethanol and purified by crystallization from hot ethanol. The final product was a yellow solid with a yield of 60% a melting point of 276 °C and purity of 99.6% (according to HPLC, Fig. S6). ^1^H NMR (500 MHz, DMSO) δ 11.13 (s, 1H), 8.81 (s, 1H), 8.40 (s, 1H), 8.35 (d, *J* = 8.6 Hz, 1H), 7.99 (d, *J* = 8.4 Hz, 1H), 7.94 (d, *J* = 7.3 Hz, 1H), 7.86 (t, *J* = 12.8 Hz, 2H), 7.76 (d, *J* = 8.4 Hz, 3H), 7.55 (t, *J* = 7.5 Hz, 1H), 7.44 (d, *J* = 16.3 Hz, 1H), 7.22 (d, *J* = 8.5 Hz, 2H), 3.38 (s, 3H), 3.21 (s, 3H). ^13^C NMR (126 MHz, DMSO) δ 162.02, 157.29, 156.28, 155.88, 152.02, 150.78, 148.27, 136.77, 134.24, 133.47, 130.14, 129.13, 128.83, 128.17, 127.47, 126.43, 121.70, 120.35, 86.88, 29.67, 27.90.

The ESI-MS that was calcd for C_24_H_21_N_5_O_2_ 412.1768 [M + H]^+^ was 412.1772.

### Biological properties

#### Cell culture

The human colon carcinoma cell line HCT 116 was obtained from ATCC. The normal human fibroblast cell line NHDF was obtained from PromoCell. The cells were grown according to previously reported method^39^ as monolayer cultures in 75 cm^2^ flasks (Nunc) in Dulbecco’s modified Eagle’s medium with the 1% v/v of penicillin/streptomycin (Gibco). The DMEM for HCT116 was supplemented with 12% heat-inactivated fetal bovine serum (Sigma) and for NHDF with 15% non-inactivated fetal bovine serum (Sigma). The cells were cultured under standard conditions at 37 °C in a humidified atmosphere at 5% CO_2_.

#### Cytotoxicity studies

These procedures were performed according to the methods reported formerly in^8,9,39,40^. In short: the cells were seeded in 96-wells plates (Nunc) (density of 4,000 and 5,000 cells/well (for NHDF and HCT 116 respectively). After incubation at 37 °C for 24 h. The assay was performed following a 72-h incubation with varying concentrations of the compound that was being tested. Then, 20 µL of CellTiter 96®AQueous One Solution-MTS (Promega) solution was added (with 100 µL DMEM without phenol red) and was incubated for 1 h at 37 °C. The optical densities of the samples were analyzed at 490 nm using a multi-plate reader (Synergy 4, BioTek). The results are expressed as the percentage of the control and were calculated as the inhibitory concentration (IC_50_) values (using GraphPad Prism 7). The IC_50_ parameter was defined as the compound concentration that was necessary to reduce the proliferation of the cells to 50% of the untreated control. A compound was tested in triplicate in a single experiment with each experiment being repeated three times.

#### The cellular staining

The staining experiments were performed following to method reported elsewhere^9,41^. HCT 116 and NHDF cells seeded on glass slides at a density of 3·10^5^ cells/slide were incubated at 37 °C for 24 h. Then, the medium was removed and a solution of **BC15** and **SC2** at a concentration of 25 μM was added. Additional 1 h of incubation under standard conditions was suitable for dye penetration. Then, the cells after being washed three times with PBS were fixed with paraformaldehyde (3.7%, 10 min.). The cellular staining with the compound being tested was observed using a Nikon Eclipse Ni-U microscope equipped with a Nikon Digital DS-Fi1-U3 camera and the corresponding software (Nikon, Tokyo, Japan).

#### Subcellular localization

For subcellular localization a known methods were applied according to^42^. The HCT 116 cells were seeded as in *2*.*3*.*3*. Then, the medium was removed and a solution of **BC15** at a concentration of 25 μM was added and the cells were further incubated for 1 h. After incubation, the cells were rinsed with PBS (pH 7.2) and the staining procedures were performed according to the provider’s protocols. Briefly, a serum-free medium that contained MitoTracker® Orange (100 nM, 30 min incubation, Molecular Probes), ER-Tracker™ Red BODIPY® TR Glibenclamide (1 µM, 30 min incubation, Molecular Probes), LysoTracker® Red DND-99 (500 nM, 1 h incubation, Molecular Probes), CellLight® Golgi-RFP, BacMam 2.0 (2 µL per 10,000 cells, 16 h incubation, Molecular Probes), CellLight® Early Endosomes-RFP, BacMam 2.0 (2 µL per 10,000 cells, 16 h incubation, Molecular Probes) were then added. After staining with organelle-specific trackers, the cells were washed three times with PBS and fixed with 3.7% paraformaldehyde for 10 minutes. The subcellular localization was observed using a Nikon Eclipse Ni-U microscope equipped with a Nikon Digital DS-Fi1-U3 camera with corresponding software (Nikon, Tokyo, Japan). The images were analyzed and processed using an Image J 1.41 (Wayne Rasband, National Institutes of Health, Bethesda, MD, USA). The Manders’ and Pearson’s coefficients, which were used to show the co-localization **BC15** with specific-organelle trackers, were calculated using the Image J plugin “JACoP”.

#### Photobleaching studies

The HCT 116 were seeded as in *2*.*3*.*3* Then, the medium was removed and a solution of **BC15** at a concentration of 25 μM was added and incubated for 2 h under standard conditions at 37 °C in a humidified atmosphere at 5% CO_2_. After incubation, the cells were washed three times with PBS and maintained in a DMEM medium without FBS and phenol red. The images were acquired during 60 minutes under 365 nm laser illumination (25% of power laser) using an inverted fluorescence microscope - Zeiss Axio Observer.Z1 equipped with AxioCam MRm camera. The relative fluorescence intensities were obtained from two selected square regions using a ImageJ 1.41 software (Wayne Rasband, National Institutes of Health, Bethesda, MD, USA).

### Spectroscopic studies

The absorption and fluorescence spectra were measured at room temperature in a 10 mm quartz cell using a U-2900 spectrophotometer (Hitachi) and an F-7000 spectrofluorimeter (Hitachi), respectively. Due to the low solubility of the compounds in the solvents that were applied, the solutions that were used to test o the f solvatochromic behavior contained 1% of DMSO in order to improve their solubility. The quantum yields of fluorescence were determined according to ref.^41^ using the absolute method at room temperature with an integrating sphere with the solvent as the blank. The compounds were excited at the wavelength that corresponded to the absorption wavelength of the compound.

### Theoretical calculations

The electronic properties of the investigated compound in the ground and excited states were calculated within the DFT and TD-DFT method with the Gaussian 09 software package^43^ with the CAM-B3LYP exchange-correlation functional^44–46^ and a 6-311 + G (d,p) basis set. The solvent effects were evaluated according to the PCM model^47^ where the cavity was created by series of overlapping spheres having the standard dielectric constants (ε) of 4.9 for chloroform. The lowest 50 singlet-singlet vertical electronic excitations, which were based on the CAM-B3LYP optimized geometries and the optimization of the first excited states, were obtained using the time-dependent density functional theory (TD-DFT) formalism^48^. The visualization of the molecular orbitals and analysis of the energy levels were performed using Chemissian software.

## Supplementary information

supplementary information
