# Supplementary material for: Acid selective pro-dye for cellular compartments
Source: Sci Rep. 2019 Oct 25;9:15304. doi: 10.1038/s41598-019-50734-8 (PMC6814713; doi:10.1038/s41598-019-50734-8)
Supplement: Supplementary file 1 — supplementary information [file 41598_2019_50734_MOESM1_ESM.docx]

**Supplementary information materials**

**Acid selective pro-dye for cellular compartments**

Barbara Czaplińska,^[a]^ Katarzyna Malarz,^[b]^ Anna Mrozek-Wilczkiewicz,^[b]^, Robert Musiol ^[a]*^

^[a]^ Institute of Chemistry, University of Silesia in Katowice, Szkolna 9, 40-007, Katowice, Poland

^[b]^ A. Chełkowski Institute of Physics and Silesian Center for Education and Interdisciplinary Research, University of Silesia in Katowice, 75 Pułku Piechoty 1, 41-500, Chorzów, Poland


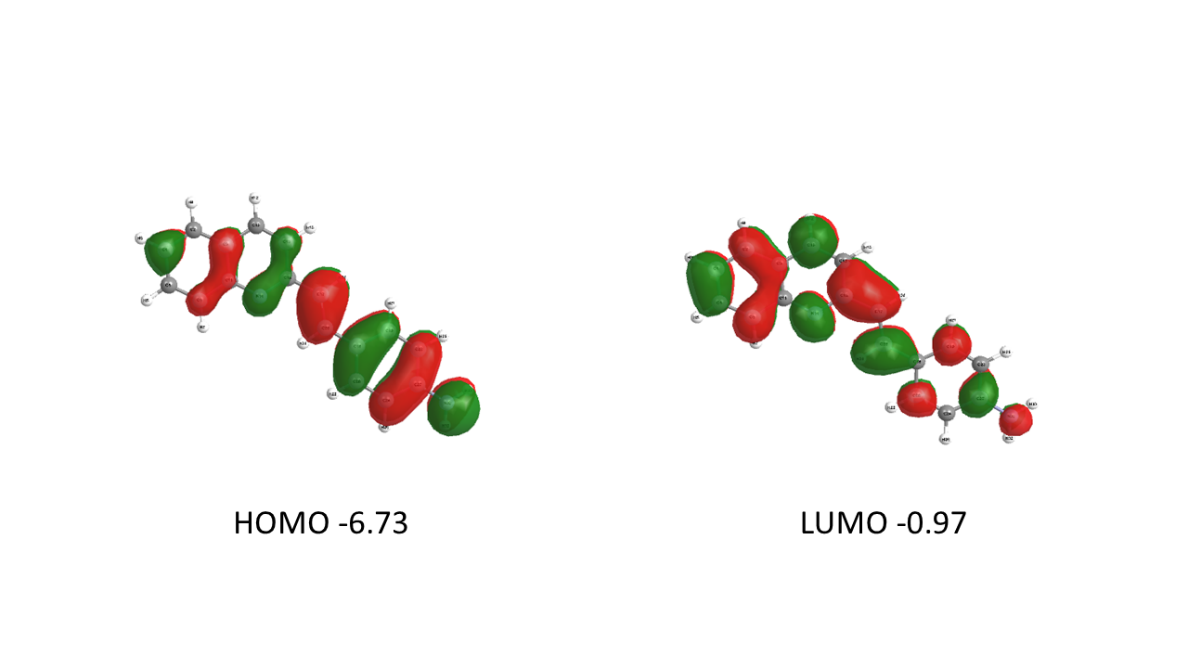


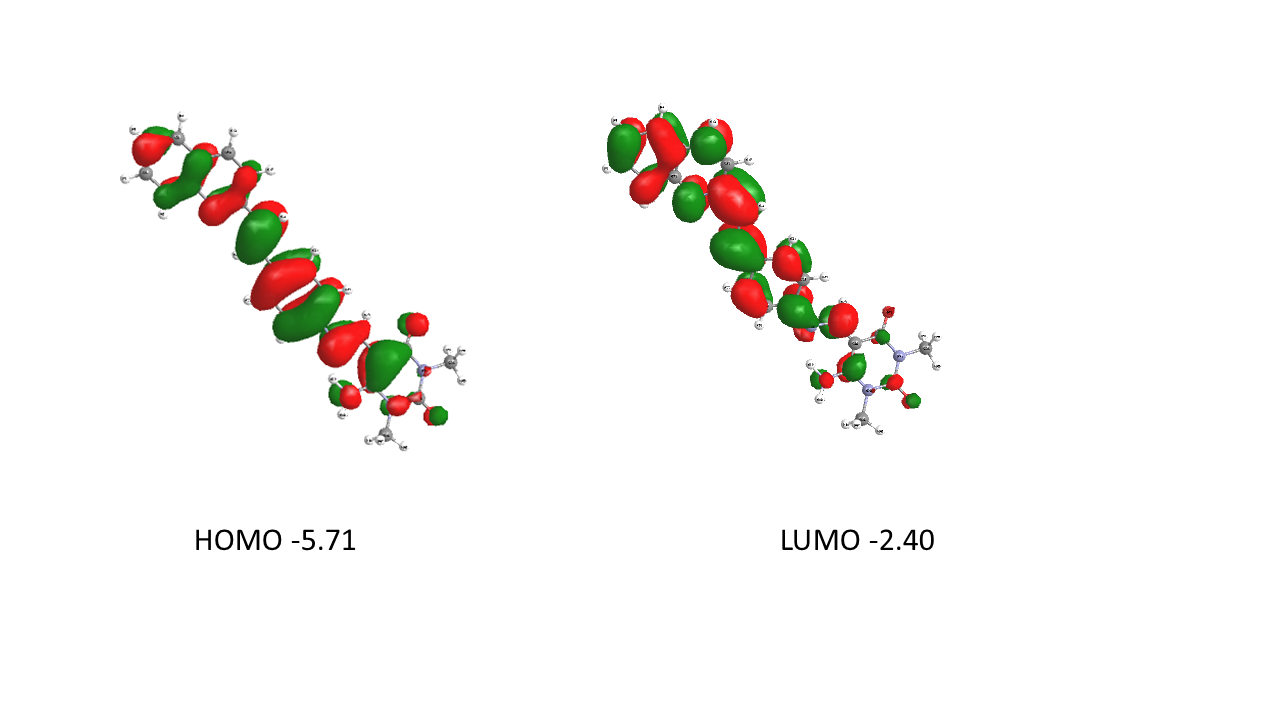


Figure S1. Contours and energies of HOMO and LUMO of compound **SC2** and **BC15.** Electronic transitions data and orbitals obtained by TD-DFT//CAM-B3LYP//6-311+G (d,p) using a PCM model (solvent – chloroform) for **SC2** at the DFT optimized geometry.

Table S1. Parameters of solvents used to describe solvatochromic properties of tested compounds. π – polarizability; α - Kamlet-Taft’s solvatochromic parameter related to hydrogen-bond donating ability; β - Kamlet-Taft’s solvatochromic parameter related to hydrogen-bond accepting ability

| solvent | Snyder polarity index | Kamlet – Taft solvents parameters | | |
| --- | --- | --- | --- | --- |
|  |  | **π** | **β** | **α** |
| toluene | **2.3** | **-** | **-** | **-** |
| chloroform | **4.4** | **0.58** | **0** | **0.44** |
| acetonitrile | **6.2** | **0.75** | **0.19** | **0.35** |
| ethanol | **5.2** | **0.54** | **0.77** | **0.83** |
| methanol | **6.6** | **0.6** | **0.62** | **0.93** |
| DMSO | **6.5** | **1** | **0.76** | **0** |

Figure S2. The plot presenting a rate of decomposition of **BC15** in water environment.

Table S2. Antiproliferative activity on the HCT 116 and NHDF cell lines.

| **compound** | **IC_50_ [µM]** | |
| --- | --- | --- |
|  | HCT 116 | NHDF |
| **BC15** | >25 | >25 |
| **SC2** | >25 | >25 |


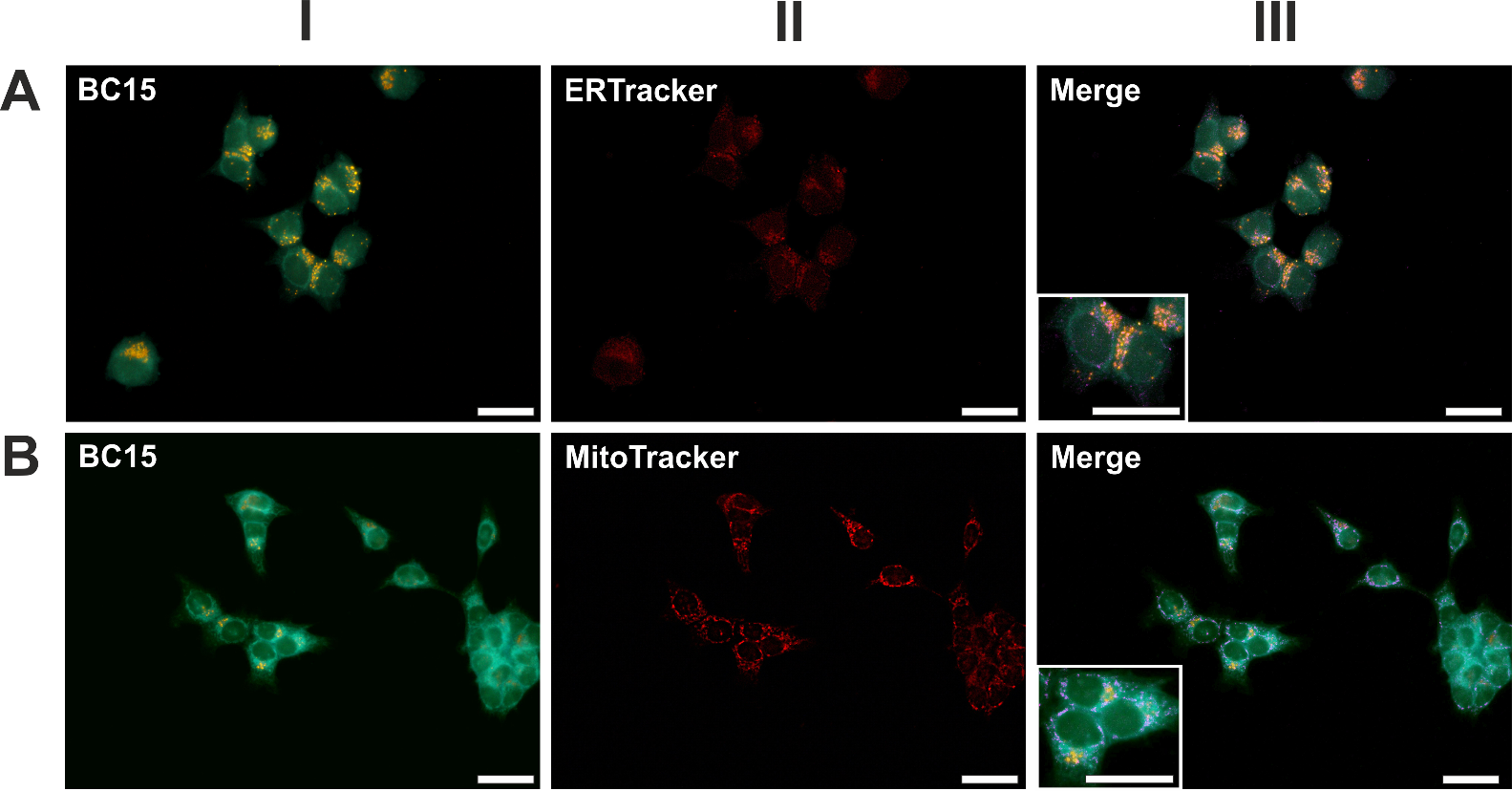


Figure S3. Fluorescence images of HCT 116 cells incubated with **BC15** (25 µM) for 2 h at 37 °C. **BC15** was excited by a DAPI - UV-2A filter (excitation wavelength 330–380 nm, barrier filter BA420). The images showed co-localization with specific-organelle trackers: endoplasmic reticulum (A) and mitochondria (B). First panel – fluorescence of the **BC15** alone, middle panel - colocalization with red tracker, last panel - merge (red-orange indicates colocalization of **BC15** with specific-organelle tracker, magenta indicates area where no colocalization was observed).). Scale bars indicate 50 µm.

Table S3. Correlation coefficients of co-localization images: protonated product of **BC15** with specific-organelle trackers.

|  | Lysosome  pH 4.5 | Endosome  pH 5.0-5.5 | Golgi Apparatus  pH 6.0-6.7 | Endoplasmic reticulum  pH 7.2 | Mitochodrium  pH 7.8 |
| --- | --- | --- | --- | --- | --- |
| Pearson’s | 0.694 | 0.731 | 0.371 | 0.352 | 0.124 |
| Manders’ | 0.938 | 0.982 | 0.353 | 0.206 | 0.073 |


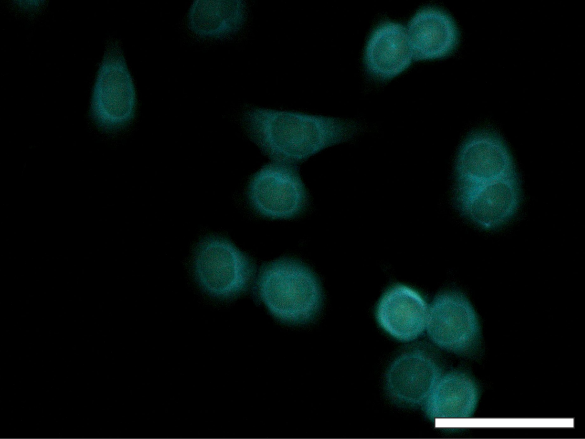


Figure S4. Fluorescence image of HCT 116 cells incubated with the compound **SC2** (25 µM) for 2 h at 37 °C. **SC2** was excited by a DAPI filter (330–380 nm) and B-2A filter (450–490 nm). Scale bars indicate 50 µm. Indistinct cell borders and other organelles indicate poor accumulation of the compound in the cell.


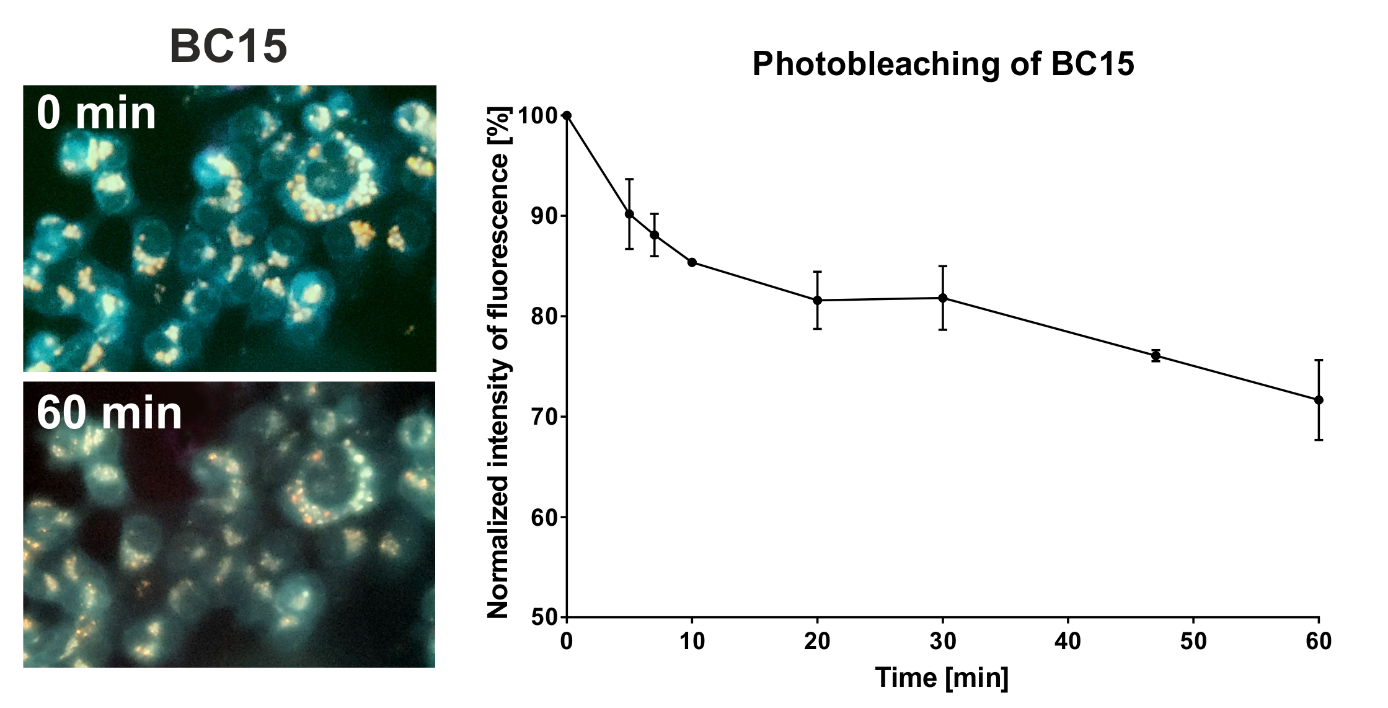


Figure S5. Photobleaching of **BC15** compound during 60 min incubation under 365 nm laser (25 % of power laser). The relative intensity of fluorescence were obtained from two selected regions in ImageJ 1.41 software.


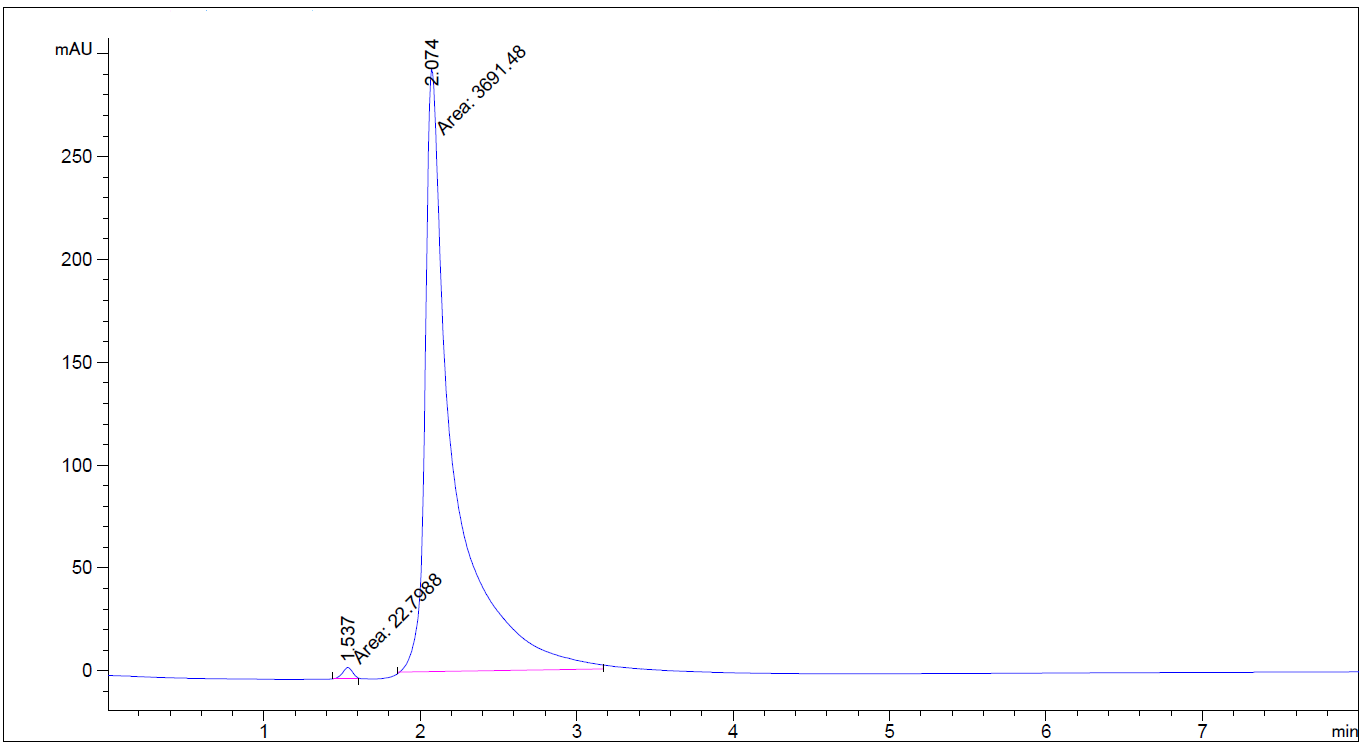


Figure S6. HPLC chromatogram of the compound BC15.

**
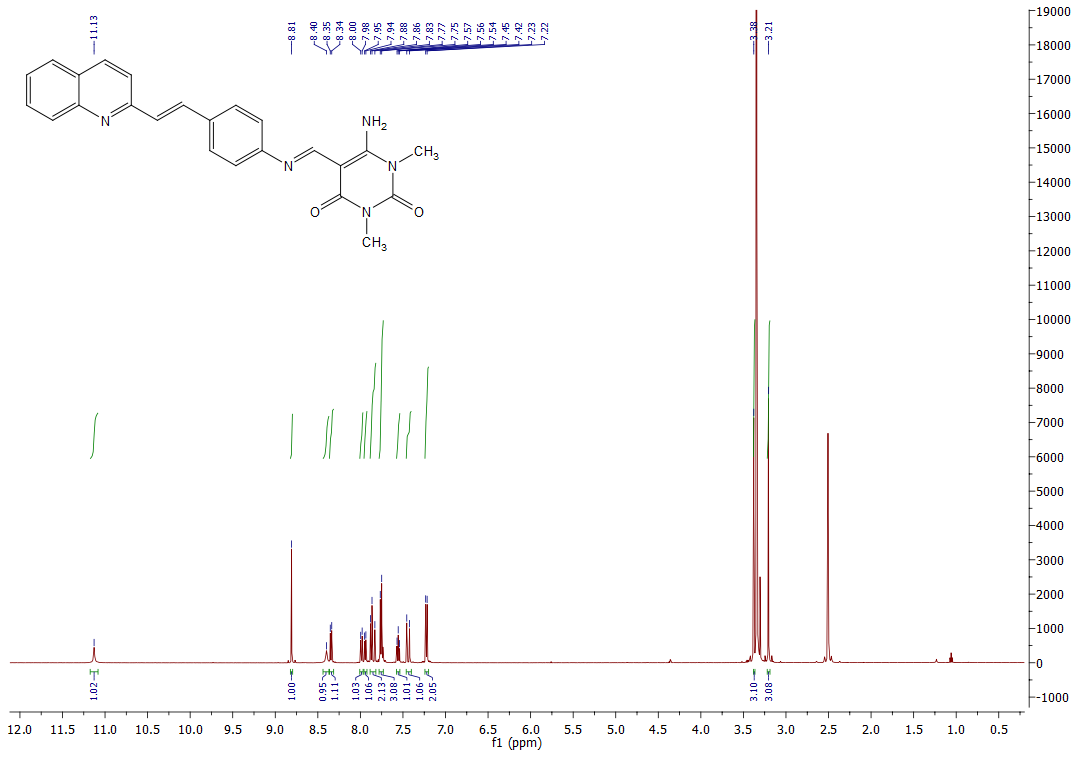
**


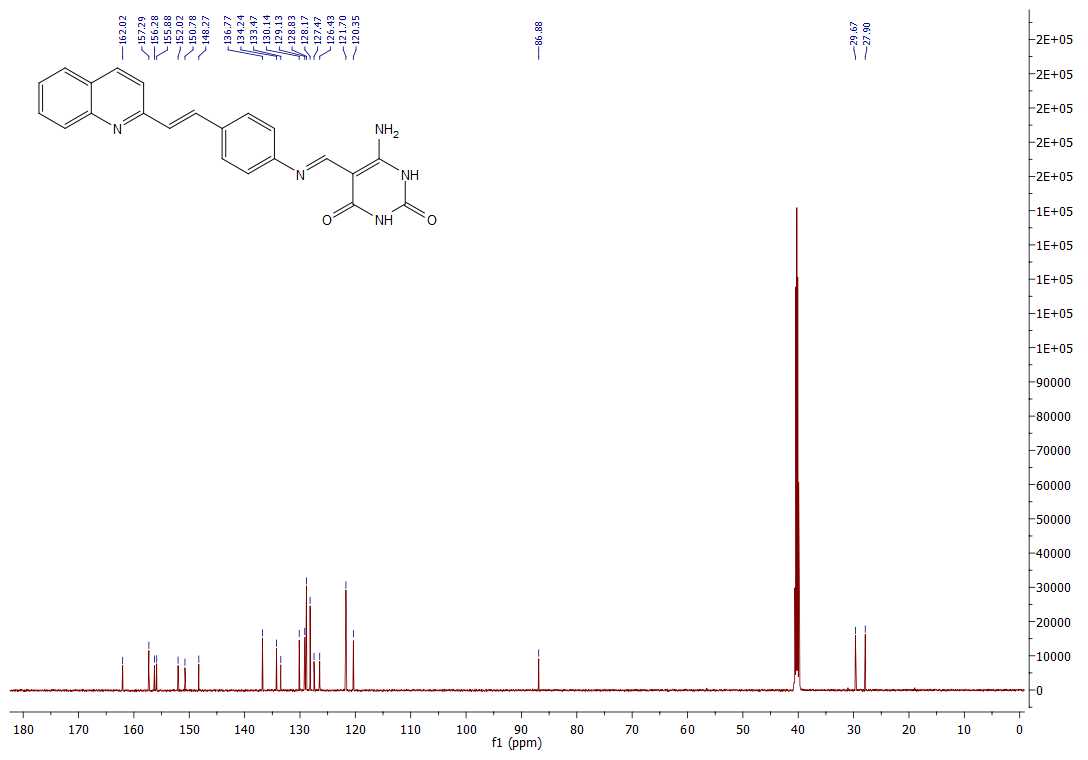


Figure S7. ^1^HNMR and ^13^CNMR spectra of the compound BC15.


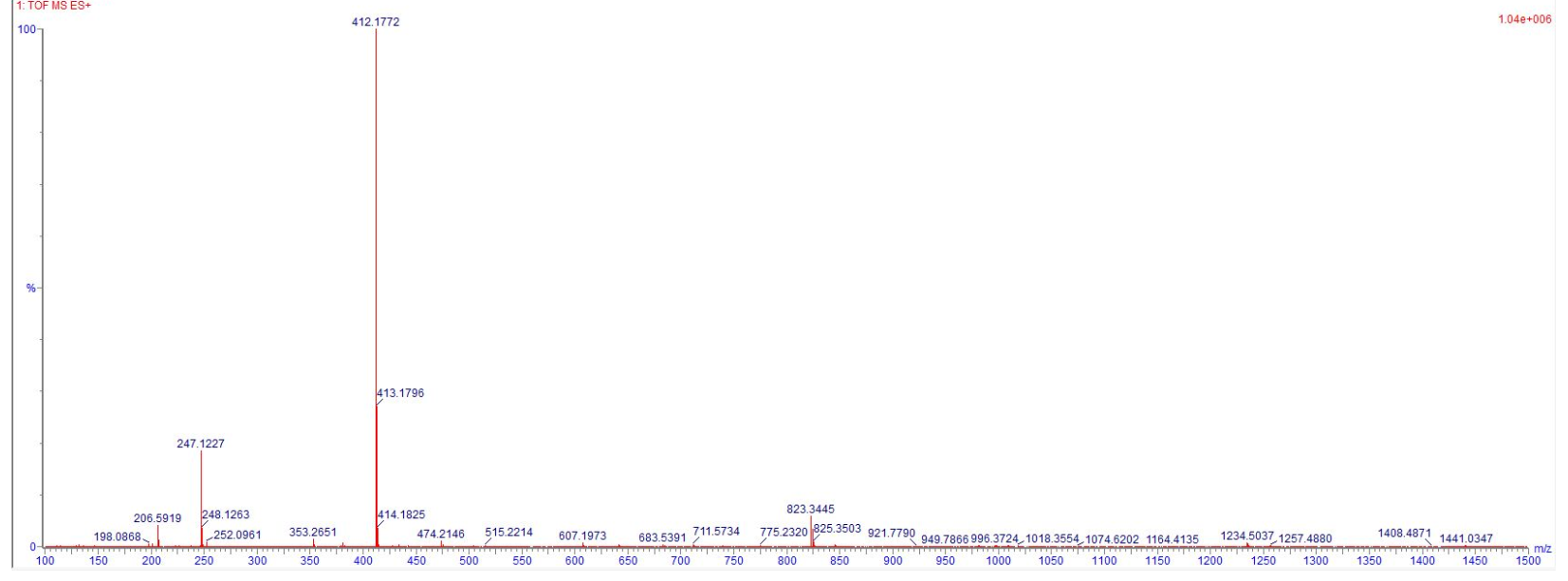


Figure S8. ESI-MS spectrum of the compound BC15.


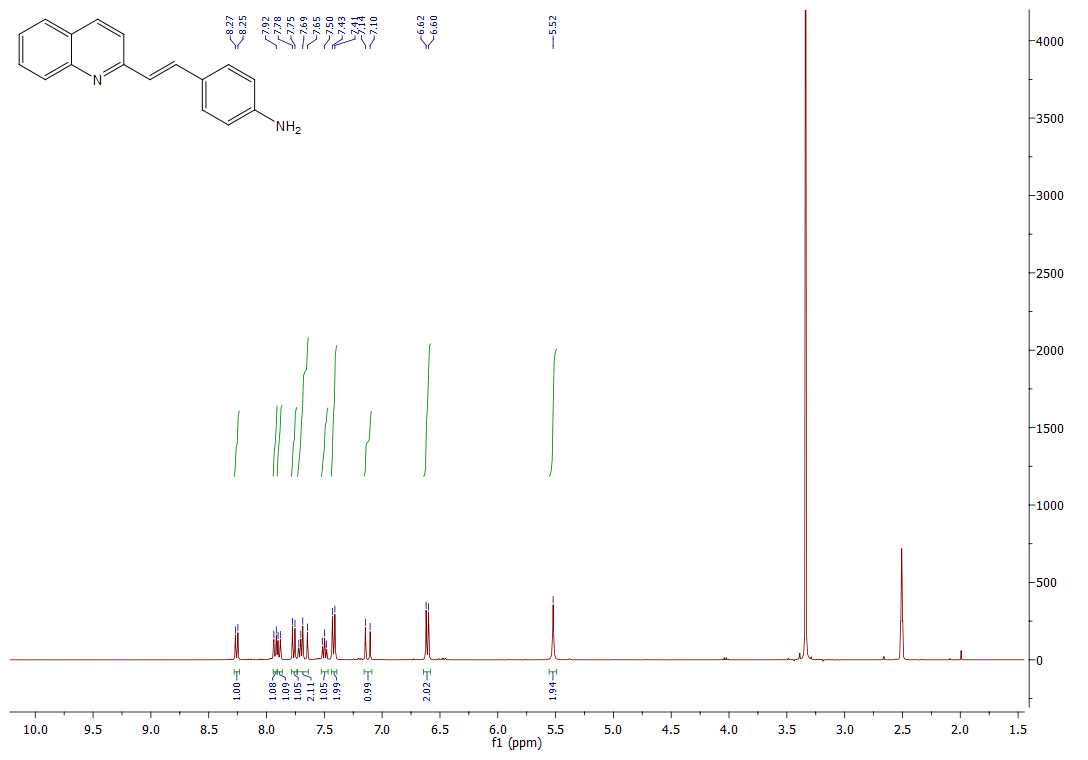


Figure S9. ^1^HNMR and ^13^CNMR spectra of the compound SC2.
